# Comparison of long-term outcomes and quality of life following radiofrequency ablation, endovenous laser ablation, and N-butyl cyanoacrylate treatment of greater saphenous vein insufficiency

**DOI:** 10.1016/j.jvsv.2025.102316

**Published:** 2025-09-12

**Authors:** Hasan Toz, Yusuf Kuserli

**Affiliations:** Department of Cardiovascular Surgery, Bakirkoy Dr. Sadi Konuk Training & Research Hospital, Istanbul, Turkey

**Keywords:** Chronic venous insufficiency, Radiofrequency ablation, Endovenous laser ablation, N-butyl cyanoacrylate, Complications, Vein occlusion

## Abstract

**Objective:**

To compare the long-term clinical outcomes and quality of life after radiofrequency ablation (RFA), endovenous laser ablation (EVLA), and N-butyl cyanoacrylate (NBCA) treatments in patients with chronic venous insufficiency.

**Methods:**

This retrospective study included 600 patients treated with RFA, EVLA, or NBCA for chronic venous insufficiency at a single center between February 2015 and February 2025. Patients were divided into 3 groups of 200 according to the treatment modality. Clinical and procedural parameters, complication rates, pain scores, time to return to daily activities, Venous Clinical Severity Scores (VCSS), and great saphenous vein (GSV) occlusion rates were compared among groups.

**Results:**

Statistically significant differences were found among the groups regarding complication rates, pain scores at 6 hours, procedure duration, time to return to daily activities, long-term GSV occlusion, and 5-year VCSS values (all *P* < .05). Complication-free rates were highest in the RFA group (89.5%), followed by NBCA (86.0%), and were lowest in the EVLA group (69.0%) (*P* < .001). EVLA had more frequent pigmentation, paresthesia, and phlebitis. NBCA had the shortest procedure time (13.7 minutes) and the greatest postprocedural pain and delayed return to daily activities. At 5 years, RFA showed the highest GSV occlusion rate (88.4%), whereas NBCA and EVLA had lower rates (70.6% and 75.0%, respectively) (*P* < .001). VCSS values at 5 years were more favorable in the RFA and NBCA groups compared with the EVLA group (*P* = .036).

**Conclusions:**

All three endovenous techniques are effective and safe for the treatment of GSV insufficiency. However, the choice of modality should consider differences in complication rates, patient comfort, and long-term vein occlusion outcomes.

**Article Highlights**

• Radiofrequency ablation showed the highest long-term great saphenous vein occlusion rate (88.4%) after 5 years.
• Endovenous laser ablation had the highest complication rates, particularly pigmentation, paresthesia, and phlebitis.
• N-butyl cyanoacrylate (NBCA) provided the shortest procedure time, but was associated with higher early pain scores and a delayed return to daily activities.
• Radiofrequency ablation and NBCA demonstrated more favorable venous clinical severity scores compared with endovenous laser ablation in long-term follow-up.

Chronic venous insufficiency (CVI) is a common and progressive circulatory system disorder characterized by inadequate venous return in the lower extremities owing to structural or functional abnormalities of the venous system.^1^ This condition, which is a significant cause of morbidity in the community, decreases quality of life of individuals and imposes an economic burden on the health care system. Pathophysiological mechanisms such as venous hypertension, valvular incompetence, and venous stasis constitute the clinical sequelae of the disease.^2^

Clinically, CVI manifests with symptoms such as edema, varicose veins, pain, skin pigmentation changes, and, in advanced cases, venous ulcers in the lower extremities.^3^ These symptoms negatively affect patients' daily living activities, leading to a decrease in functional capacity.^4^ Therefore, the implementation of effective and long-lasting treatment approaches is of great importance, both for achieving symptomatic improvement and for preventing complications.

Today, minimally invasive endovenous treatment methods have come to the forefront as alternatives to surgical treatment options.^5^ Radiofrequency ablation (RFA), endovenous laser ablation (EVLA), and N-butyl cyanoacrylate (NBCA) injection are among the frequently used methods in the treatment of saphenous vein insufficiency.^6^, ^7^, ^8^ These techniques are preferred owing to advantages such as shorter hospital stays, superior cosmetic outcomes, rapid recovery, and lower complication rates.^9^ However, comparative evaluation of these methods is needed, especially regarding their long-term clinical efficacy and effects on patients' quality of life.

The aim of this study was to comparatively evaluate the long-term clinical outcomes and quality of life effects of RFA, EVLA, and NBCA methods used in the treatment of CVI and to demonstrate the efficacy and safety of these treatment options based on objective data.

## Methods

### Study design and duration

This study retrospective, cross-sectional, single-center observational study was conducted at the Department of Cardiovascular Surgery, Bakırköy Dr. Sadi Konuk Training and Research Hospital, between February 2015 and February 2025. Over this 10-year period, the clinical outcomes and quality-of-life data for patients treated with three different endovenous ablation methods for CVI were analyzed comparatively.

### Patient selection and grouping

A total of 600 patients who were treated with RFA, EVLA, or NBCA injection for great saphenous vein (GSV) or small saphenous vein insufficiency were included in the study. Three groups were formed with 200 patients in each group, corresponding with each treatment modality. The age range of the participants was 20 to 65 years, their clinical classifications varied between C2 and C5 according to the Clinical-Etiological-Anatomical-Pathophysiological (CEAP) classification system.

### Inclusion and exclusion criteria

In patients included in the study, color Doppler ultrasound (CDUS) examination performed in the outpatient setting detected saphenous veins with reflux lasting >0.5 seconds and a diameter of ≥5 mm in women and ≥5.5 mm in men, located 2 cm distal to the saphenofemoral or saphenopopliteal junction. Patients with CEAP stages C2 to C5 and at ≥5 years of follow-up data were included. Patients were excluded if they had tortuous vascular anatomy, a history of deep vein thrombosis or pulmonary embolism, active thrombophlebitis, previous surgical varicose vein intervention, pregnancy, immobilization, an aneurysm >15 mm in the target vein, local or systemic infection, or known hypersensitivity to cyanoacrylate.

### Applied procedures

The diagnoses of patients for whom endovenous treatment was planned owing to venous insufficiency were confirmed by CDUS examination of the lower extremity venous system. During the assessment, patients with a GSV diameter of ≥5 mm in women and ≥5.5 mm in men and a reflux duration of >4 seconds, as well as without significant vessel tortuosity, were included in the study. In this study, a total of 600 patients were included and equally divided into three groups: 200 patients underwent RFA, 200 patients underwent EVLA, and 200 patients underwent NBCA injection, all as unilateral procedures. Because the number of eligible patients in each modality exceeded 200, patients fulfilling the inclusion criteria were selected randomly to obtain equal group sizes and ensure comparability across treatment arms. Cases with a GSV diameter of >10 mm at the knee level were not considered eligible for endovenous treatment. Additionally, individuals with a history of deep vein thrombosis (DVT), active venous ulcer, a history of phlebitis, prior venous surgery, or a diagnosis of lymphedema or lipedema were excluded from the study according to the exclusion criteria.

#### RFA application protocol

Before the RFA procedure and after the patients were taken to the operating room, areas of venous distension were identified and marked while the patient was in the standing position. Spinal anesthesia was administered to all patients; in 10 patients where sufficient analgesia could not be achieved during the procedure, airway control was provided with the support of a laryngeal mask. Following antiseptic preparation and sterilization, a 7F sheath was inserted into the GSV using the Seldinger technique under CDUS guidance, most commonly from the below-knee level, but in some cases from the knee or above. The RFA catheter was then positioned approximately 2 cm distal to the saphenofemoral junction. For tumescent anesthesia, a mixture was prepared by adding 60 mL of 8.4% sodium bicarbonate, 30 mL of 2% prilocaine, and 0.6 mg adrenaline to 1000 mL of isotonic solution at +4°C, and injected along the GSV from the sheath level up to the saphenofemoral junction. During ablation, RFA was performed by applying 120°C heat for about 20 seconds to each 5-cm segment.

#### EVLA application protocol

During the EVLA procedure, a 7F sheath was inserted into the GSV below the level of the knee under Doppler ultrasound guidance. A single-use, 12 W radial laser fiber with a wavelength of 1470 nm (EVLAS Circular-2; FG Group) was advanced approximately 2 cm distal to the saphenofemoral junction. For tumescent anesthesia, a solution was prepared by adding 5 mg bupivacaine, 0.6 mg adrenaline, and 7 ampules of 8.4% sodium bicarbonate to 1000 mL of isotonic solution cooled to 4°C, and this mixture was injected around the GSV from the sheath level up to the saphenofemoral junction. During laser application, the probe was gradually withdrawn, delivering 60 to 75 J/cm (average of 70 J/cm) of energy over approximately 5 seconds for each 1-cm segment to achieve ablation. Throughout the procedure, manual compression was applied externally along the target vein to collapse the vessel and ensure effective thermal ablation.

#### NBCA ablation application protocol

The NBCA ablation procedure was initiated by placing a 7F sheath into the GSV at or below the level of the knee under ultrasonographic guidance. In all cases, Invamed VenaBLOCK (Invamed Sağlık İlaç Sanayi ve Ticaret A.Ş.) was used as the embolic agent. The tip of the catheter was positioned approximately 3 cm distal to the saphenofemoral junction. Before the procedure, patients were placed in the Trendelenburg position to reduce venous distension, and external compression was applied to the saphenofemoral junction using the ultrasound probe. The cyanoacrylate adhesive was then injected along the GSV over approximately 30 seconds to achieve ablation. During the withdrawal of the catheter, simultaneous external compression was applied over the GSV. After the adhesion was completed, external compression was maintained for an average of 4 to 5 minutes. After the procedure, the effectiveness of the intervention was confirmed by CDUS examination through the assessment of reduced GSV diameter and increased echogenicity within the vessel lumen.

### Follow-up protocol

Patients were mobilized within the first 6 hours after surgery, and visual analog pain scale scores were recorded. After dressing change on the first postoperative day, patients began using elastic compression stockings. The patency of the treated vein was evaluated by CDUS examination at the 1-, 3-, and 6-month, as well as 1-, 2-, 3-, and 5-year postoperative follow-up visits. At each follow-up, the CEAP classification, Venous Clinical Severity Score (VCSS), and postoperative complications were also recorded. All 600 patients were evaluated throughout the follow-up period.

### Ethical approval

The study was approved by the Clinical Research Ethics Committee of Bakırköy Dr. Sadi Konuk Training and Research Hospital, University of Health Sciences, on April 24, 2025, with decision number 2025/138. The purpose of the study was explained to all participants, and written informed consent was obtained. The research was conducted in full compliance with the principles of the Declaration of Helsinki and relevant regulations.

### Statistical analyses

All statistical analyses were performed using SPSS version 27.0 software (IBM). The distribution of the data was evaluated using the Kolmogorov-Smirnov test for normality. Continuous variables were expressed as mean ± standard deviation, and categorical variables were presented as numbers and percentages. Comparisons of continuous variables between more than two groups were conducted using one-way analysis of variance for normally distributed data and the Kruskal-Wallis test for non-normally distributed data. Post hoc pairwise comparisons were performed using the Tukey test or Dunn's test as appropriate. The chi-square test was used to compare categorical variables. A *P* value of <.05 was considered statistically significant.

An a priori power analysis was performed using G∗Power software (version 3.1.9.7) to determine the required sample size for the study. A one-way analysis of variance (fixed effects, omnibus, one-way) was selected as the appropriate statistical method to compare three independent treatment groups. Based on existing literature, an effect size of *f* = 0.25 (medium effect) was assumed. The significance level (α) was set at 0.05, and the desired statistical power (1 – β) was set at 0.90. The analysis indicated that a minimum total sample size of 207 participants (approximately 69 per group) would be sufficient to detect a statistically significant difference among the groups under these parameters. However, considering the retrospective design of the study and the availability of clinical data, a total of 600 patients (200 per group) were ultimately included in the analysis. This sample size exceeds the calculated minimum requirement, thereby ensuring sufficient statistical power to support the validity and reliability of the study findings.

## Results

A statistically significant difference was observed in the complication rates among patients treated with RFA, EVLA, and NBCA (*P* < .001). The rate of patients without complications was highest in the RFA group: 89.5% (n = 179). This rate was 86.0% (n = 172) in the NBCA group and significantly lower in the EVLA group: 69.0% (n = 138). Notably, the frequency of complications was significantly higher in the EVLA group. When the types of complications were examined, pigmentation was observed in 10 patients (5.0%), paresthesia in 12 patients (6.0%), phlebitis in 13 patients (6.5%), and DVT in 4 patients (2.0%) in the EVLA group, which were higher compared with the other treatment groups. The rate of DVT in the NBCA group was 3.0% (n = 6), whereas no DVT cases were observed in the RFA group. Pigmentation, paresthesia, and phlebitis were also more frequently encountered in the EVLA group compared with the other groups, and this difference was found to be statistically significant (*P* < .001) (Table I).

**Table I:** Comparison of sex, Clinical-Etiological-Anatomical-Pathophysiological (*CEAP*) classification, side distribution, and complications in patients treated with radiofrequency ablation (*RFA*), endovenous laser ablation (*EVLA*), and N-butyl cyanoacrylate (*NBCA*)

	RFA (n = 200)	EVLA (n = 200)	NBCA (n = 200)	*P* value
Sex				
Female	99 (49.5)	101 (50.5)	97 (48.5)	.923
Male	101 (50.5)	99 (49.5)	103 (51.5)	
CEAP classification				
C2	129 (64.5)	124 (62.0)	130 (65.0)	.152
C2r	2 (1.0)	11 (5.5)	2 (1.0)	
C3	54 (27.0)	49 (24.5)	55 (27.5)	
C4a	8 (4.0)	7 (3.5)	6 (3.0)	
C4b	7 (3.5)	9 (4.5)	7 (3.5)	
Side				
Right	103 (51.5)	105 (52.5)	105 (52.5)	.661
Left	77 (38.5)	83 (41.5)	77 (38.5)	
Bilateral	20 (10.0)	12 (6.0)	18 (9.0)	
Complications				
No	179 (89.5)	138 (69.0)	172 (86.0)	**<.001**
Pigmentation	3 (1.5)	10 (5.0)	5 (2.5)	
Paresthesia	2 (1.0)	12 (6.0)	8 (4.0)	
Phlebitis	6 (3.0)	13 (6.5)	8 (4.0)	
DVT	0 (0.0)	4 (2.0)	6 (3.0)	
Hematoma	3 (1.5)	7 (3.5)	0 (0.0)	
Ecchymosis	4 (2.0)	10 (5.0)	1 (0.5)	
Burn	3 (1.5)	6 (3.0)	0 (0.0)	

*DVT,* Deep vein thrombosis.

Values are number (%). Boldface entries indicate statistical significance.

When the RFA, EVLA, and NBCA groups were compared, the shortest procedure time was observed in the NBCA group (13.7 ± 1.1 minutes), and the longest was measured in the EVLA group (32.2 ± 4.4 minutes; *P* < .001). The 6-hour postprocedural VAS scores were significantly higher in the NBCA group (4.9 ± 0.5), whereas they were 2.3 ± 0.8 and 2.5 ± 1.0 in the RFA and EVLA groups, respectively (*P* < .001). Regarding the time to return to daily activities, this period was significantly longer in the NBCA group with 2.2 ± 0.5 days compared with the other groups. In the RFA and EVLA groups, the time to return to daily life was 1.6 ± 0.3 days (*P* < .001) (Table II).

**Table II:** Comparison of demographic, procedural, and clinical parameters among the three different treatment groups

	RFA (n = 200)	EVLA (n = 200)	NBCA (n = 200)	*P* value
Age, years	46.5 ± 11.3	45.0 ± 11.7	44.0 ± 11.9	.113
BMI, kg/m^2^	26.2 ± 3.2	26.4 ± 3.2	26.0 ± 2.8	.519
GSV diameter, mm	7.5 ± 1.9	7.7 ± 2.0	6.8 ± 1.0	**<.001**
Procedure duration, minutes	26.6 ± 4.4	32.2 ± 4.4	13.7 ± 1.1	**<.001**
Preprocedural VAS	8.8 ± 0.7	8.9 ± 0.7	8.8 ± 0.7	.124
VAS at 6 hours postprocedure	2.3 ± 0.8	2.5 ± 1.0	4.9 ± 0.5	**<.001**
Time to return to daily activities, days	1.6 ± 0.3	1.6 ± 0.3	2.2 ± 0.5	**<.001**

*BMI,* Body mass index; *EVLA,* endovenous laser ablation; *GSV,* great saphenous vein; *RFA,* radiofrequency ablation; *NBCA,* N-butyl cyanoacrylate; *VAS,* visual analog scale.

Values are mean ± standard deviation. Boldface entries indicate statistical significance.

No statistically significant differences were found between the groups in terms of preprocedural, first month, sixth month, first year, and third year mean VCSS values (*P* = .146, *P* = .241, *P* = .172, *P* = .894, and *P* = .110, respectively). However, a statistically significant difference was observed between the groups at the end of year 5. The 5-year VCSS value was 6.3 ± 1.7 in the RFA group, 6.6 ± 1.5 in the EVLA group, and 6.3 ± 1.7 in the NBCA group (*P* = .036) (Table III).

**Table III:** Comparison of Venous Clinical Severity Scores (*VCSS*) values over time among three different treatment groups

	RFA (n = 200)	EVLA (n = 200)	NBCA (n = 200)	*P* value
Preprocedural VCSS	18.4 ± 3.7	18.4 ± 2.8	19.0 ± 3.5	.146
VCSS at 1 month postprocedure	13.9 ± 2.5	13.8 ± 2.1	14.2 ± 2.3	.241
VCSS at 6 months postprocedure	11.1 ± 1.7	11.0 ± 1.4	11.2 ± 1.6	.172
VCSS at 1 year postprocedure	9.2 ± 1.6	9.2 ± 1.2	9.2 ± 1.5	.894
VCSS at 3 years postprocedure	7.7 ± 1.6	7.9 ± 1.3	7.8 ± 1.5	.110
VCSS at 5 years postprocedure	6.3 ± 1.7	6.6 ± 1.5	6.3 ± 1.7	**.036**

*EVLA,* Endovenous laser ablation; *RFA,* radiofrequency ablation; *NBCA,* N-butyl cyanoacrylate.

Values are mean ± standard deviation. Boldface entries indicate statistical significance.

No significant difference was observed between the groups in GSV occlusion rates at the end of the first month (RFA, 100.0 ± 0.0%; EVLA, 99.9 ± 0.5%; NBCA, 99.9 ± 0.4%; *P* = .762). At month 3, the GSV occlusion rate was 99.3 ± 1.6% in the RFA group, 96.9 ± 1.6% in the EVLA group, and 96.1 ± 2.1% in the NBCA group, and this difference was found to be statistically significant (*P* = .036). At the 6-month, 12-month, 2-year, 3-year, and 5-year follow-ups, significant differences in GSV occlusion rates were observed between the groups (all *P* < .001). At month 6, the occlusion rates were 98.8 ± 2.0% in the RFA group, 94.4 ± 2.1% in the EVLA group, and 91.0 ± 1.7% in the NBCA group. At month 12, these rates were 96.6 ± 3.0%, 92.5 ± 2.1%, and 87.4 ± 1.9%, respectively. As the follow-up period advanced, a decrease in GSV occlusion rates was observed in all groups; however, at the end of year 5, the occlusion rate was 88.4 ± 3.4% in the RFA group, 75.0 ± 1.9% in the EVLA group, and 70.6 ± 0.9% in the NBCA group, with a significant difference between the groups (*P* < .001) (Table IV).

**Table IV:** Comparison of great saphenous vein (*GSV*) occlusion rates over time among the three different treatment groups

	RFA (n = 200)	EVLA (n = 200)	NBCA (n = 200)	*P* value
GSV occlusion rate at 1 month postoperative	100.0 ± 0.0	99.9 ± 0.5	99.9 ± 0.4	.762
GSV occlusion rate at 3 months postoperative	99.3 ± 1.6	96.9 ± 1.6	96.1 ± 2.1	**.036**
GSV occlusion rate at 6 months postoperative e	98.8 ± 2.0	94.4 ± 2.1	91.0 ± 1.7	**<.001**
GSV occlusion rate at 12 months postoperative	96.6 ± 3.0	92.5 ± 2.1	87.4 ± 1.9	**<.001**
GSV occlusion rate at 2 years postoperative	93.9 ± 3.7	90.7 ± 2.1	84.3 ± 1.4	**<.001**
GSV occlusion rate at 3 years postoperative	91.6 ± 3.6	85.3 ± 2.0	80.5 ± 1.6	**<.001**
GSV occlusion rate at 5 years postoperative	88.4 ± 3.4	75.0 ± 1.9	70.6 ± 0.9	**<.001**

*EVLA,* Endovenous laser ablation; *RFA,* radiofrequency ablation; *NBCA,* N-butyl cyanoacrylate.

Values are mean ± standard deviation. Boldface entries indicate statistical significance.

## Discussion

In this study, three different endovenous methods—RFA, EVLA, and NBCA—were compared in the treatment of CVI with respect to various clinical and procedural parameters. The findings revealed statistically significant differences among the groups, particularly in complication rates, early postoperative pain levels, time to return to daily activities, GSV occlusion rates, and long-term clinical scores. Analysis of complication distribution showed that certain types of complications were more frequent in specific groups, with one group being notably more disadvantaged in terms of complication incidence. Additionally, early postoperative pain and time to resume daily life varied depending on the treatment modality, whereas long-term follow-up revealed significant differences among the groups in both clinical scores and saphenous vein occlusion rates. These results suggest that the short- and long-term efficacy and safety profiles of the treatment methods may differ.

In our study, significant differences were observed among the RFA, EVLA, and NBCA groups in terms of both complication rates and procedural and clinical outcomes. Our findings demonstrated that the incidence of complications and postoperative pain was notably higher in the EVLA group compared with the other methods, whereas NBCA had the shortest procedure duration. In the long-term follow-up, the occlusion success rates of RFA and NBCA were found to be higher than that of EVLA. These results are consistent with the study by El Kılıç et al,^8^ which similarly reported higher complication and pain scores in the EVLA group and a significantly shorter procedure time for NBCA. Moreover, both studies showed that although early occlusion rates were comparable across all groups, RFA achieved particularly higher success in the long term compared with EVLA. In the study by El Kılıç et al, NBCA and RFA demonstrated similar long-term efficacy, whereas EVLA was highlighted as being less advantageous in terms of both complication rate and long-term occlusion success. In a study by Bahadır et al,^10^ it was shown that RFA provided the highest long-term occlusion rates and patient comfort among endovenous treatment options, whereas EVLA and NBCA embolization demonstrated relatively lower long-term vein patency rates and a higher incidence of recanalization over 5 years.

The reported complication rates after the treatment of CVI have shown substantial variation, largely owing to inconsistencies in defining complications—specifically, whether pain is included as a complication. This lack of standardization has contributed to conflicting findings across the literature. For instance, Çalık et al^11^ evaluated both EVLA and NBCA in the management of CVI, comparing pain scores and time to resumption of daily activities, but did not assess the overall complication rate. Their results demonstrated significantly more favorable outcomes with NBCA. Similarly, Morrison et al^12^ observed that both NBCA and RFA yielded comparable rates of complications and pain levels. Gibson et al^13^ also found no significant differences in total complication rates between NBCA and RFA procedures. In contrast, the current study identified a markedly higher complication rate in the EVLA group.

Although the occlusion rates among NBCA, RFA, and EVLA were found to be similar, NBCA demonstrated superior patient comfort compared with the other techniques. Both EVLA and RFA have shown highly satisfactory occlusion rates in the literature, with EVLA achieving rates between 92% and 100% during short- and mid-term follow-up periods, as previously reported.^14^, ^15^, ^16^, ^17^ Similarly, RFA has been associated with short- and mid-term occlusion rates ranging from 90% to 100%.^18^, ^19^, ^20^, ^21^ NBCA has also yielded promising short-term occlusion rates, reported between 90.3% and 100% in earlier studies,^7^, ^8^, ^9^, ^10^ with a 30-month occlusion rate of 94.1% observed in one investigation.^22^ Nevertheless, it should be noted that these findings were primarily derived from retrospective analyses. To date, only two prospective studies have directly compared EVLA and NBCA, both of which reported no statistically significant differences between the groups.^23^^,^^24^ Furthermore, the VeClose trial remains the sole randomized clinical trial directly comparing NBCA with RFA.^25^^,^^26^ In the randomized clinical trial by Eroğlu and Yasim,^27^ no significant differences were found among NBCA, RFA, and EVLA regarding 2-year vein closure and complication rates, whereas NBCA was associated with a shorter procedure time and greater patient comfort. These findings are generally consistent with our study. However, in our cohort, early postoperative pain levels were higher and the time to return to daily activities was longer in the NBCA group, which may be related to differences in patient profile or procedural techniques. In conclusion, both studies indicate that long-term success is comparable among the methods, while early postoperative patient comfort and procedure duration may vary depending on the technique.

In an study by Er et al,^28^ EVLA and NBCA were found to have similar saphenous vein occlusion rates, although patient satisfaction was reported to be significantly higher in the NBCA group. Similarly, in our study, there was no notable difference between the two methods in terms of early and long-term vein closure success. However, improved procedural comfort, greater patient satisfaction, and earlier return to daily activities in patients treated with NBCA support the advantages in patient experience reported in the literature. These results suggest that patient satisfaction and comfort may be important determinants in the selection of minimally invasive treatment options.

In a review by Chan et al (2021)^5^ evaluating the efficacy and safety of endovenous NBCA ablation in the Asian population, it was emphasized that the NBCA technique provided high patient satisfaction, a low complication rate, and high anatomical success in the short term, along with a significant improvement in quality of life. The study noted that NBCA was not inferior to traditional endothermal methods in terms of anatomical success and complication profile, and even offered clear advantages regarding patient comfort and procedural pain. In our study as well, high early occlusion rates were achieved in the NBCA group; however, a significant decrease in occlusion rates was observed over time, and long-term results were lower compared with RFA. Nevertheless, as highlighted by Chan et al, the technical ease of NBCA application and the generally low postoperative pain observed in patients are consistent with our findings that NBCA had the shortest procedure time and was generally a safe option in terms of its complication profile.

In a study by Koramaz et al,^24^ NBCA and EVLA were found to provide similar anatomical and clinical success, but NBCA was associated with a shorter procedure time and a lower complication rate. In our study as well, NBCA stood out with its short procedure time and low complication profile, and clinical improvement was observed to be similar for both methods. Sincos et al,^6^ in contrast, compared RFA with surgical treatment and found no difference in long-term vein closure rates or clinical outcomes, but demonstrated that RFA offered advantages with shorter recovery times and hospital stays. Overall, these results are consistent with our findings and support that minimally invasive endovenous treatments are advantageous both in terms of efficacy and patient comfort.

This study has several limitations that should be acknowledged. First, the single-center design and the relatively limited sample size may affect the generalizability of our findings. Second, the follow-up period, although sufficient for short- and mid-term outcomes, was not long enough to assess long-term efficacy and potential late complications. Third, as with most studies in this field, patient-reported outcomes such as comfort and satisfaction were subject to potential reporting bias. Additionally, although efforts were made to minimize confounding factors, the retrospective nature of the data collection may have introduced selection bias. Finally, the lack of blinding in treatment allocation could have influenced both clinical assessments and patient-reported outcomes. Further multicenter, prospective, randomized controlled trials with larger sample sizes and extended follow-up periods are needed to validate these findings. Finally, although routine duplex ultrasound examination was performed preoperatively to exclude acute deep vein thrombosis, a detailed evaluation of deep venous valve competence or reflux was not performed. This factor represents a limitation of our study.

## Conclusions

All three endovenous treatment modalities—NBCA, RFA, and EVLA—demonstrate high efficacy in the management of CVI, with comparable occlusion rates observed among the groups. However, differences in patient comfort and complication profiles may influence the selection of the optimal treatment approach. In our study, RFA showed more favorable outcomes compared with EVLA across multiple parameters, suggesting that RFA may represent a more advantageous option in terms of long-term efficacy and safety. Nevertheless, EVLA remains a widely used and guideline-endorsed modality, and treatment selection should be individualized based on patient characteristics, resource availability, and physician expertise. Further prospective, randomized studies with larger cohorts and longer follow-up periods are required to better define the relative advantages and long-term outcomes of these techniques.

## Author contributions

Conception and design: HT

Analysis and interpretation: YK

Data collection: Not applicable

Writing the article: HT

Critical revision of the article: HT, YK

Final approval of the article: HT, YK

Statistical analysis: YK

Obtained funding: Not applicable

Overall responsibility: HT

## Funding

None.

## Disclosures

None.
